# Supplementary material for: A computational modelling framework to quantify the effects of passaging cell lines
Source: PLoS One. 2017 Jul 27;12(7):e0181941. doi: 10.1371/journal.pone.0181941 (PMC5531485; doi:10.1371/journal.pone.0181941)
Supplement: S1 Supporting Information — (PDF) [file pone.0181941.s001.pdf]

PLOS ONE

# Supporting Information:

# A computational modelling framework to quantify the effects of passaging cell lines

**Wang Jin · Catherine J Penington ·**

**Scott W McCue · Matthew J Simpson**

July 13, 2017

School of Mathematical Sciences, Queensland University of Technology (QUT), Brisbane, Australia

E-mail: matthew.simpson@qut.edu.au

### C++ code for cell culture growth

```cpp
#include <cmath>
#include <iostream>
#include <vector>
#include <stdio.h>
#include <stdlib.h>
#include <fstream>
#include <ctime>
#include <string>
#include <sstream>
#include <algorithm>
#include <iterator>
#include <random>

using namespace std;

void migration1 (const int agent_row, const int agent_col,
                 const int rowN, const int colN, int * migPosition) {
    migPosition[0] = agent_row;
    migPosition[1] = agent_col;
    double P = ((double) rand() / (RAND_MAX)); //choose migration directions
    if ((P < 1.0/6.0) && (agent_row > 0) && (agent_col > 0)) {//Position 1
        migPosition[0] = migPosition[0] - 1;
        migPosition[1] = migPosition[1] - 1;}
    else if ((P >= 1.0/6.0) && (P <1.0/3.0) && (agent_row > 0) && (agent_col < colN-1)) {//Position 2
        migPosition[0] = migPosition[0] - 1;}
    else if ((P >= 1.0/3.0) && (P < 1.0/2.0) && (agent_col < colN-1)) {//Position 3
        migPosition[1] = migPosition[1] + 1;}
    else if ((P >= 1.0/2.0) && (P < 2.0/3.0) && (agent_row < rowN-1) && (agent_col < colN-1)) {//Position 4
        migPosition[0] = migPosition[0] + 1;}
    else if ((P >= 2.0/3.0) && (P < 5.0/6.0) && (agent_row < rowN-1) && (agent_col > 0)) {//Position 5
        migPosition[0] = migPosition[0] + 1;
        migPosition[1] = migPosition[1] - 1;}
    else if ((P >= 5.0/6.0) && (agent_col > 0)) {//Position 6
        migPosition[1] = migPosition[1] - 1;}
}
```

```

void migration2 (const int agent_row, const int agent_col,
                 const int rowN, const int colN, int * migPosition) {
    migPosition[0] = agent_row;
    migPosition[1] = agent_col;
    double P = ((double) rand() / (RAND_MAX)); //choose migration directions
    if ((P < 1.0/6.0) && (agent_row > 0)) {//Position 1
        migPosition[0] = migPosition[0] - 1;}
    else if ((P >= 1.0/6.0) && (P <1.0/3.0) && (agent_row > 0) && (agent_col < colN-1)) {//Position 2
        migPosition[0] = migPosition[0] - 1;
        migPosition[1] = migPosition[1] + 1;}
    else if ((P >= 1.0/3.0) && (P < 1.0/2.0) && (agent_col < colN-2)) {//Position 3
        migPosition[1] = migPosition[1] + 1;}
    else if ((P >= 1.0/2.0) && (P < 2.0/3.0) && (agent_row < rowN-1) && (agent_col < colN-1)) {//Position 4
        migPosition[0] = migPosition[0] + 1;
        migPosition[1] = migPosition[1] + 1;}
    else if ((P >= 2.0/3.0) && (P < 5.0/6.0) && (agent_row < rowN-1)) {//Position 5
        migPosition[0] = migPosition[0] + 1;}
    else if ((P >= 5.0/6.0) && (agent_col > 0)) {//Position 6
        migPosition[1] = migPosition[1] - 1;}
}

void proliferation1 (const int agent_row, const int agent_col,
                     const int rowN, const int colN, int * proPosition) {
    proPosition[0] = agent_row;
    proPosition[1] = agent_col;
    double P = ((double) rand() / (RAND_MAX)); //choose proliferation directions
    if ((P < 1.0/6.0) && (agent_row > 0) && (agent_col > 0)) {//Position 1
        proPosition[0] = proPosition[0] - 1;
        proPosition[1] = proPosition[1] - 1;}
    else if ((P >= 1.0/6.0) && (P <1.0/3.0) && (agent_row > 0) && (agent_col < colN-1)) {//Position 2
        proPosition[0] = proPosition[0] - 1;}
    else if ((P >= 1.0/3.0) && (P < 1.0/2.0) && (agent_col < colN-1)) {//Position 3
        proPosition[1] = proPosition[1] + 1;}
```

```
    else if ((P >= 1.0/2.0) && (P < 2.0/3.0) && (agent_row < rowN-1) && (agent_col < colN-1)) {//Position 4
        proPosition[0] = proPosition[0] + 1;}
    else if ((P >= 2.0/3.0) && (P < 5.0/6.0) && (agent_row < rowN-1) && (agent_col > 0)) {//Position 5
        proPosition[0] = proPosition[0] + 1;
        proPosition[1] = proPosition[1] - 1;}
    else if ((P >= 5.0/6.0) && (agent_col > 0)) {//Position 6
        proPosition[1] = proPosition[1] - 1;}
}

void proliferation2 (const int agent_row, const int agent_col,
                     const int rowN, const int colN, int * proPosition) {
    proPosition[0] = agent_row;
    proPosition[1] = agent_col;
    double P = ((double) rand() / (RAND_MAX)); //choose proliferation directions
    if ((P < 1.0/6.0) && (agent_row > 0)) {//Position 1
        proPosition[0] = proPosition[0] - 1;}
    else if ((P >= 1.0/6.0) && (P <1.0/3.0) && (agent_row > 0) && (agent_col < colN-1)) {//Position 2
        proPosition[0] = proPosition[0] - 1;
        proPosition[1] = proPosition[1] + 1;}
    else if ((P >= 1.0/3.0) && (P < 1.0/2.0) && (agent_col < colN-2)) {//Position 3
        proPosition[1] = proPosition[1] + 1;}
    else if ((P >= 1.0/2.0) && (P < 2.0/3.0) && (agent_row < rowN-1) && (agent_col < colN-1)) {//Position 4
        proPosition[0] = proPosition[0] + 1;
        proPosition[1] = proPosition[1] + 1;}
    else if ((P >= 2.0/3.0) && (P < 5.0/6.0) && (agent_row < rowN-1)) {//Position 5
        proPosition[0] = proPosition[0] + 1;}
    else if ((P >= 5.0/6.0) && (agent_col > 0)) {//Position 6
        proPosition[1] = proPosition[1] - 1;}
}

int CalTotal (const int rowN, const int colN) {
    int Ntotal;
    if (rowN % 2 == 0) {
        Ntotal = rowN * colN - rowN/2;
    } else {
```

```
        Ntotal = rowN * colN - (rowN+1)/2;
    }
    return Ntotal;
}

int main(int argc, char **argv) {
    srand(time(NULL)); //random seeds
    random_device rd;
    mt19937 generator(rd());
    //set clock
    clock_t TIME;
    TIME = clock();

    //Initialisation
    int passageI, passageN, t, tau;
    double Pm, Pp;
    passageI = 0; //passage index
    passageN = 30; //max passage number
    tau = 1; //time step: 1 ~ 1/12h
    t = 0; //current time
    const int d = 24; //spacing: 1 ~ 24mum
    enum {rowN = 3610, colN = 4168}; //full scale
    //enum {rowN = 362, colN = 418}; //scale ~ 1/10
    const int Ntotal = CalTotal(rowN,colN);
    const int Nstart = Ntotal * 0.15; //seeding condition
    const int Nend = Ntotal * 0.85; //85% confluent
    static vector<vector<int> > domain(rowN, std::vector<int>(colN)); //simulation domain
    static vector<vector<double> > domain_Pp(rowN, std::vector<double>(colN)); //Pp
    //static vector<vector<int> > domain_passage(rowN, std::vector<int>(colN)); //passage
    //vector<vector<int> > domain_age(rowN, std::vector<int>(colN)); //age
    //static vector<vector<int> > domain_generation(rowN, std::vector<int>(colN)); //generation
    Pm = 0.35; //migration probability
    //default_random_engine generator;
    normal_distribution<double> distribution(4e-3,1e-3); //Pp distribution
    normal_distribution<double> eps1(2e-5,2e-5); //eps1 distribution
    normal_distribution<double> eps2(1e-5,1e-6); //eps2 distribution
    int passageEff = 0;
    int ageEff = 0;
```

```
    double Pd = 1.0;
    double Pd2 = 0.0;
    double avePp;

    ofstream file1;
    ofstream file2;
    //ofstream file3;
    //ofstream file4;
    //ofstream file5;
    ofstream file6;
    ofstream file7;
    //ofstream file8;
    //ofstream file9;
    //ofstream file10;
    ofstream file11;
    file1.open("domain.csv");
    file2.open("domain_Pp.csv");
    //file3.open("domain_passage.csv");
    //file4.open("domain_age.csv");
    //file5.open("domain_generation.csv");
    file6.open("domain2.csv");
    file7.open("domain2_Pp.csv");
    //file8.open("domain2_passage.csv");
    //file9.open("domain2_age.csv");
    //file10.open("domain2_generation.csv");
    file11.open("td.csv");

    //Seeding
    int agent_row, agent_col;
    for (int i=Nstart; i>0; --i) {
        agent_row = rand() % rowN;
        agent_col = rand() % colN;
        if (((rowN-agent_row) % 2 != 0) && (agent_col == colN-1)) {
            i = i + 1;
        } else if (domain[agent_row][agent_col] == 0) {
            domain[agent_row][agent_col] = 1;
            domain_Pp[agent_row][agent_col] = distribution(generator);
            if (domain_Pp[agent_row][agent_col] < 0) {
                domain_Pp[agent_row][agent_col] = 0.0;
            }

        } else {
```

```
            i = i + 1;
        }
    }

    //Count initial cells
    int agent_N = 0;
    for (int i=0; i<rowN; ++i) {
        for (int j=0; j<colN; ++j) {
            if (domain[i][j] == 1) {
                agent_N = agent_N + 1;
            }
        }
    }
    //cout << agent_N;

    //Write to files
    for (int i=0; i<rowN; ++i) {
        for (int j=0; j<colN; ++j) {
            file1 << domain[i][j];
            file2 << domain_Pp[i][j];
            //file3 << domain_passage[i][j];
            //file4 << domain_age[i][j];
            //file5 << domain_generation[i][j];
            if (j < colN-1) {
                file1 << ", ";
                file2 << ", ";
                //file3 << ", ";
                //file4 << ", ";
                //file5 << ", ";
            } else {
                file1 << "\n";
                file2 << "\n";
                //file3 << "\n";
                //file4 << "\n";
                //file5 << "\n";
            }
        }
    }

    t = t + tau;
    int migPosition[2], proPosition[2];
    double P;
```

```

    while (passageI <= passageN) {//check passage number
        cout << "passage number #" << passageI << ", ";
        int N = agent_N;
        cout << "N(0) = " << N << endl;

        //check average Pp
        avePp = 0;
        for (int i=0; i<rowN; ++i) {
            for (int j=0; j<colN; ++j) {
                if (domain[i][j] == 1) {
                    avePp = avePp + domain_Pp[i][j];
                }
            }
        }
        avePp = avePp/N;
        cout << avePp << endl;
        while (N < Nend) { //check cell population
            //Migration
            for (int i=0; i<N; ++i) {
                P = ((double) rand() / (RAND_MAX)); //generate a random number
                if (P <= Pm) { //perform migration
                    //select a random site
                    int ifGhost = 0;
                    agent_row = rand() % rowN;
                    agent_col = rand() % colN;
                    while ((domain[agent_row][agent_col] != 1) || (ifGhost == 1)) { //select a random site, stop until finding an agent
                        agent_row = rand() % rowN;
                        agent_col = rand() % colN;
                    }
                    //test ghost node
                    if (((rowN-agent_row) % 2 != 0) && (agent_col == colN-1)) {
                        ifGhost = 1;
                    }
                    if (ifGhost == 0) {
                        if ((rowN-agent_row) % 2 == 0) {
                            migration1(agent_row, agent_col, rowN, colN, migPosition);
```

```
                if (domain[migPosition[0]][migPosition[1]] == 0) {//if the new site is not taken
                    domain[agent_row][agent_col] = 0; //delete the previous site
                    domain[migPosition[0]][migPosition[1]] = 1; //move to the new site
                    /*if (domain_Pp[migPosition[0]][migPosition[1]]!= 0 && domain_passage[migPosition[0]][migPosition[1]]!= 0 && domain_age[migPosition[0]][migPosition[1]]!= 0 && domain_generation[migPosition[0]][migPosition[1]]!= 0) {
                    cout << "error mig 1" << endl;
                    }*/
                    domain_Pp[migPosition[0]][migPosition[1]] = domain_Pp[agent_row][agent_col]; //move the corresponding Pp
                    //domain_passage[migPosition[0]][migPosition[1]] = domain_passage[agent_row][agent_col]; //move the corresponding passage index
                    //domain_age[migPosition[0]][migPosition[1]] = domain_age[agent_row][agent_col]; //move the corresponding age
                    //domain_generation[migPosition[0]][migPosition[1]] = domain_generation[agent_row][agent_col]; //move the corresponding generation
                    domain_Pp[agent_row][agent_col] = 0.0; //delete the previous Pp
                    //domain_passage[agent_row][agent_col] = 0; //delete the previous passage index
                    //domain_age[agent_row][agent_col] = 0; //delete the previous age
                    //domain_generation[agent_row][agent_col] = 0; //delete the previous generation
                    /*if (domain_Pp[agent_row][agent_col]!=0 || domain_passage[agent_row][agent_col]!=0 || domain_age[agent_row][agent_col]!=0 || domain_generation[agent_row][agent_col]!=0) {
```

```
                        cout << "error mig 1" << endl;
                    }*/
                }
            } else {
                migration2(agent_row, agent_col, rowN, colN, migPosition);
                if (domain[migPosition[0]][migPosition[1]] == 0) {//if the new site is not taken
                    domain[agent_row][agent_col] = 0; //delete the previous site
                    domain[migPosition[0]][migPosition[1]] = 1; //move to the new site
                    /*if (domain_Pp[migPosition[0]][migPosition[1]]!= 0 && domain_passage[migPosition[0]][migPosition[1]]!= 0 && domain_age[migPosition[0]][migPosition[1]]!= 0 && domain_generation[migPosition[0]][migPosition[1]]!= 0) {
                        cout << "error mig 2" << endl;
                    }*/
                    domain_Pp[migPosition[0]][migPosition[1]] = domain_Pp[agent_row][agent_col]; //move the corresponding Pp
                    //domain_passage[migPosition[0]][migPosition[1]] = domain_passage[agent_row][agent_col]; ////move the corresponding passage index
                    //domain_age[migPosition[0]][migPosition[1]] = domain_age[agent_row][agent_col]; //move the corresponding age
                    //domain_generation[migPosition[0]][migPosition[1]] = domain_generation[agent_row][agent_col]; //move the corresponding generation
                    domain_Pp[agent_row][agent_col] = 0.0; //delete the previous Pp
                    //domain_passage[agent_row][agent_col] = 0; //delete the previous passage index
                    //domain_age[agent_row][agent_col] = 0; //delete the previous age
```

```
                                //domain_generation[agent_row][agent_col] = 0; //delete the previous age
                                /*if (domain_Pp[agent_row][agent_col]!=0 || domain_passage[agent_row][agent_col]!=0 || domain_age[agent_row][agent_col]!=0 || domain_generation[agent_row][agent_col]!=0) {
                                    cout << "error mig 2" << endl;
                                }*/
                            }
                        }
                    } else {
                        i = i - 1;
                    }

                }
            }
            //Proliferation
            for (int i=0; i<N; ++i) {
                //select a random site
                int ifGhost = 0;
                agent_row = rand() % rowN;
                agent_col = rand() % colN;
                while ((domain[agent_row][agent_col] != 1) || (ifGhost == 1)) { //select a random site, stop until finding an agent
                    agent_row = rand() % rowN;
                    agent_col = rand() % colN;
                }
                //test ghost node
                if (((rowN-agent_row) % 2 != 0) && (agent_col == colN-1)) {
                    ifGhost = 1;
                }
                if (ifGhost == 0)
                {
                    Pp = domain_Pp[agent_row][agent_col];
                    P = ((double) rand() / (RAND_MAX)); //generate random number
                    if (P <= Pp) {
                        if ((rowN-agent_row) % 2 == 0) {
```

```
                proliferation1(agent_row, agent_col, rowN, colN, proPosition);
                if (domain[proPosition[0]][proPosition[1]] == 0) {//if the new site is not taken
                    domain[proPosition[0]][proPosition[1]] = 1; //proliferate at the new site
                    P = ((double) rand() / (RAND_MAX)); //generate a random number
                    if (ageEff && P<=Pd2) {//damage
                        domain_Pp[proPosition[0]][proPosition[1]] = Pp - max(eps2(generator), 0.0);
                        domain_Pp[proPosition[0]][proPosition[1]] = max(domain_Pp[proPosition[0]][proPosition[1]], 0.0);
                        domain_Pp[agent_row][agent_col] = domain_Pp[proPosition[0]][proPosition[1]];
                        if (domain_Pp[agent_row][agent_col]<0 || domain_Pp[agent_row][agent_col]!=domain_Pp[proPosition[0]][proPosition[1]]) {
                            cout << "error" << endl;
                        }
                    } else {//no damage
                        domain_Pp[proPosition[0]][proPosition[1]] = Pp; //adopt Pp
                    }
                    //domain_passage[proPosition[0]][proPosition[1]] = passageI; //passage number when it was born
                    //domain_age[proPosition[0]][proPosition[1]] = -1; //initiate age = -1 so that when updating cell age it equals 0
                    //domain_generation[proPosition[0]][proPosition[1]] = domain_generation[agent_row][agent_col] + 1; //add genertion
                    //domain_generation[agent_row][agent_col] = domain_generation[agent_row][agent_col] + 1; //add generation
```

```
                agent_N = agent_N + 1; //update agent population
            }
        } else {
            proliferation2(agent_row, agent_col, rowN, colN, proPosition);
            if (domain[proPosition[0]][proPosition[1]] == 0) {//if the new site is not taken
                domain[proPosition[0]][proPosition[1]] = 1; //proliferate at the new site
                P = ((double) rand() / (RAND_MAX)); // generate a random number
                if (ageEff && P<=Pd2) {//damage
                    domain_Pp[proPosition[0]][proPosition[1]] = Pp - max(eps2(generator), 0.0);
                    domain_Pp[proPosition[0]][proPosition[1]] = max(domain_Pp[proPosition[0]][proPosition[1]], 0.0);
                    domain_Pp[agent_row][agent_col] = domain_Pp[proPosition[0]][proPosition[1]];
                    if (domain_Pp[agent_row][agent_col]<0 || domain_Pp[agent_row][agent_col]!=domain_Pp[proPosition[0]][proPosition[1]]) {
                        cout << "error" << endl;
                    }
                } else {//no damage
                    domain_Pp[proPosition[0]][proPosition[1]] = Pp; //adopt Pp
                }
                //domain_passage[proPosition[0]][proPosition[1]] = passageI; //passage number when it was born
                //domain_age[proPosition[0]][proPosition[1]] = -1; //initiate age = -1 so that when updating cell age it equals 0
                //domain_generation[proPosition[0]][proPosition[1]] = domain_generation[agent_row][agent_col] + 1; //add generation
```

```
                                //domain_generation[agent_row][agent_col] = domain_generation[agent_row][agent_col] + 1; //add generation
                                /*if (passageI!=0)
                                {
                                    cout << "Pp = " << Pp << endl;
                                }*/
                                agent_N = agent_N + 1; //update agent population
                            }
                        }
                    }
                } else {
                    i = i - 1;
                }
            }

            //update cell age
            /*for (int i=0; i<rowN; ++i) {
                for (int j=0; j<colN; ++j) {
                    if (domain[i][j]==1) { //check if there is a cell
                        domain_age[i][j] = domain_age[i][j] + tau;
                    }
                }
            }*/

            N = agent_N; //update cell number
            //cout << "N(" << t << ") = " << N << endl;

            //Write to files
            if (N >= Nend) {
                for (int i=0; i<rowN; ++i) {
                    for (int j=0; j<colN; ++j) {
                        file6 << domain[i][j];
                        file7 << domain_Pp[i][j];
                        //file8 << domain_passage[i][j];
                        //file9 << domain_age[i][j];
                        //file10 << domain_generation[i][j];
                        if (j < colN-1) {
                            file6 << ", ";
                            file7 << ", ";
                            //file8 << ", ";
```

```
                        //file9 << ", ";
                        //file10 << ", ";
                    } else {
                        file6 << "\n";
                        file7 << "\n";
                        //file8 << "\n";
                        //file9 << "\n";
                        //file10 << "\n";
                    }
                }
            }
        }

        t = t + tau;

    }
    //cout << " DONE N = " << agent_N << endl;
    cout << "DONE N(" << (t-tau) << ") = " << N << endl;
    file11 << (t-tau);
    if (passageI != passageN + 1)
    {
        file11 << ", ";
    }

    //check average Pp
    avePp = 0;
    for (int i=0; i<rowN; ++i) {
            for (int j=0; j<colN; ++j) {
                if (domain[i][j] == 1) {
                    avePp = avePp + domain_Pp[i][j];
                }
            }
    }
    avePp = avePp/N;
    cout << avePp << endl;

    //Subculturing
    passageI = passageI + 1; //update passage number
    t = 0; //reset time
    if (passageI != passageN + 1) {//check if subculture
        agent_N = 0; //delete current cell number
        double * Pp_temp;
```

```
            //int * passage_temp;
            //int * generation_temp;
            Pp_temp = new double [Nstart];
            //passage_temp = new int [Nstart];
            //generation_temp = new int[Nstart];
            //Select cells
            for (int i=Nstart; i>0; --i) {
                agent_row = rand() % rowN;
                agent_col = rand() % colN;
                if (((rowN-agent_row) % 2 != 0) && (agent_col == colN-1)) {
                    i = i + 1;
                } else if (domain[agent_row][agent_col] == 1) {
                                        Pp_temp[Nstart-i] = domain_Pp[agent_row][agent_col];
                                        //passage_temp[Nstart-i] = domain_passage[agent_row][agent_col];
                                        //age_temp[Nstart-i] = domain_age[agent_row][agent_col];
                                        //generation_temp[Nstart-i] = domain_generation[agent_row][agent_col];
                } else {
                    i = i + 1;
                }
            }

            //Delete cells
            for (int i=0; i<rowN; ++i) {
                for (int j=0; j<colN; ++j) {
                    if (domain[i][j] == 1) {
                        domain[i][j] = 0;
                        domain_Pp[i][j] = 0.0;
                        //domain_passage[i][j] = 0;
                        //domain_age[i][j] = 0;
                        //domain_generation[i][j] = 0;
                    }
                }
            }

                        //Shuffle & delete temp values
```

```
                        for (int i=Nstart; i>0; --i) {
                                agent_row = rand() % rowN;
                                agent_col = rand() % colN;
                                if (((rowN-agent_row) % 2 != 0) && (agent_col == colN-1)) {
                                        i = i + 1;
                                } else if (domain[agent_row][agent_col] == 0) {
                                        domain[agent_row][agent_col] = 1;
                    P = ((double) rand() / (RAND_MAX)); //generate a random number
                    if (passageEff && P<=Pd) {//damage
                        domain_Pp[agent_row][agent_col] = Pp_temp[Nstart-i] - max(eps1(generator), 0.0);
                        //cout << domain_Pp[agent_row][agent_col] << endl;
                        domain_Pp[agent_row][agent_col] = max(domain_Pp[agent_row][agent_col], 0.0);
                        if (domain_Pp[agent_row][agent_col] < 0) {
                            cout << "error" << endl;
                        }
                    } else {//no damage
                        domain_Pp[agent_row][agent_col] = Pp_temp[Nstart-i];
                    }
                    //domain_passage[agent_row][agent_col] = passage_temp[Nstart-i];
                    //domain_age[agent_row][agent_col] = age_temp[Nstart-i];
                    //domain_generation[agent_row][agent_col] = generation_temp[Nstart-i];

                                        agent_N = agent_N + 1;
                                } else {
                                        i = i + 1;
                                }
                        }

            //Delete arrays
            delete[] Pp_temp;
            //delete[] passage_temp;
            //delete[] generation_temp;

```

```
            //Write to files
            for (int i=0; i<rowN; ++i) {
                for (int j=0; j<colN; ++j) {
                    file1 << domain[i][j];
                    file2 << domain_Pp[i][j];
                    //file3 << domain_passage[i][j];
                    //file4 << domain_age[i][j];
                    //file5 << domain_generation[i][j];
                    if (j < colN-1) {
                        file1 << ", ";
                        file2 << ", ";
                        //file3 << ", ";
                        //file4 << ", ";
                        //file5 << ", ";
                    } else {
                        file1 << "\n";
                        file2 << "\n";
                        //file3 << "\n";
                        //file4 << "\n";
                        //file5 << "\n";
                    }
                }
            }
        }
    }


    file1.close();
    file2.close();
    //file3.close();
    //file4.close();
    //file5.close();
    file6.close();
    file7.close();
    //file8.close();
    //file9.close();
    //file10.close();
    file11.close();

    TIME = clock() - TIME;
    cout << "It takes "<< (float)TIME/CLOCKS_PER_SEC <<" second(s)."<<
        endl;
```

```
    cin.get();
    return 0;
}
```

**C++ code for scratch assays**

```
#include <cmath>
#include <iostream>
#include <vector>
#include <stdio.h>
#include <stdlib.h>
#include <fstream>
#include <ctime>
#include <string>
#include <sstream>
#include <algorithm>
#include <iterator>
#include <random>

using namespace std;

void migration1 (const int agent_row, const int agent_col,
                 const int rowN, const int colN, int * migPosition) {
    migPosition[0] = agent_row;
    migPosition[1] = agent_col;
    double P = ((double) rand() / (RAND_MAX)); //choose migration
        directions
    if ((P < 1.0/6.0) && (agent_row > 0) && (agent_col > 0)) {//Position 1
        migPosition[0] = migPosition[0] - 1;
        migPosition[1] = migPosition[1] - 1;}
    else if ((P >= 1.0/6.0) && (P <1.0/3.0) && (agent_row > 0) && (
        agent_col < colN-1)) {//Position 2
        migPosition[0] = migPosition[0] - 1;}
    else if ((P >= 1.0/3.0) && (P < 1.0/2.0) && (agent_col < colN-1)) {//
        Position 3
        migPosition[1] = migPosition[1] + 1;}
    else if ((P >= 1.0/2.0) && (P < 2.0/3.0) && (agent_row < rowN-1) && (
        agent_col < colN-1)) {//Position 4
        migPosition[0] = migPosition[0] + 1;}
    else if ((P >= 2.0/3.0) && (P < 5.0/6.0) && (agent_row < rowN-1) && (
        agent_col > 0)) {//Position 5
        migPosition[0] = migPosition[0] + 1;
        migPosition[1] = migPosition[1] - 1;}
    else if ((P >= 5.0/6.0) && (agent_col > 0)) {//Position 6
        migPosition[1] = migPosition[1] - 1;}
}
```

```

void migration2 (const int agent_row, const int agent_col,
                 const int rowN, const int colN, int * migPosition) {
    migPosition[0] = agent_row;
    migPosition[1] = agent_col;
    double P = ((double) rand() / (RAND_MAX)); //choose migration directions
    if ((P < 1.0/6.0) && (agent_row > 0)) {//Position 1
        migPosition[0] = migPosition[0] - 1;}
    else if ((P >= 1.0/6.0) && (P <1.0/3.0) && (agent_row > 0) && (agent_col < colN-1)) {//Position 2
        migPosition[0] = migPosition[0] - 1;
        migPosition[1] = migPosition[1] + 1;}
    else if ((P >= 1.0/3.0) && (P < 1.0/2.0) && (agent_col < colN-2)) {//Position 3
        migPosition[1] = migPosition[1] + 1;}
    else if ((P >= 1.0/2.0) && (P < 2.0/3.0) && (agent_row < rowN-1) && (agent_col < colN-1)) {//Position 4
        migPosition[0] = migPosition[0] + 1;
        migPosition[1] = migPosition[1] + 1;}
    else if ((P >= 2.0/3.0) && (P < 5.0/6.0) && (agent_row < rowN-1)) {//Position 5
        migPosition[0] = migPosition[0] + 1;}
    else if ((P >= 5.0/6.0) && (agent_col > 0)) {//Position 6
        migPosition[1] = migPosition[1] - 1;}
}

void proliferation1 (const int agent_row, const int agent_col,
                     const int rowN, const int colN, int * proPosition) {
    proPosition[0] = agent_row;
    proPosition[1] = agent_col;
    double P = ((double) rand() / (RAND_MAX)); //choose migration directions
    if ((P < 1.0/6.0) && (agent_row > 0) && (agent_col > 0)) {//Position 1
        proPosition[0] = proPosition[0] - 1;
        proPosition[1] = proPosition[1] - 1;}
    else if ((P >= 1.0/6.0) && (P <1.0/3.0) && (agent_row > 0) && (agent_col < colN-1)) {//Position 2
        proPosition[0] = proPosition[0] - 1;}
    else if ((P >= 1.0/3.0) && (P < 1.0/2.0) && (agent_col < colN-1)) {//Position 3
        proPosition[1] = proPosition[1] + 1;}
```

```
    else if ((P >= 1.0/2.0) && (P < 2.0/3.0) && (agent_row < rowN-1) && (agent_col < colN-1)) {//Position 4
        proPosition[0] = proPosition[0] + 1;}
    else if ((P >= 2.0/3.0) && (P < 5.0/6.0) && (agent_row < rowN-1) && (agent_col > 0)) {//Position 5
        proPosition[0] = proPosition[0] + 1;
        proPosition[1] = proPosition[1] - 1;}
    else if ((P >= 5.0/6.0) && (agent_col > 0)) {//Position 6
        proPosition[1] = proPosition[1] - 1;}
}

void proliferation2 (const int agent_row, const int agent_col,
                     const int rowN, const int colN, int * proPosition) {
    proPosition[0] = agent_row;
    proPosition[1] = agent_col;
    double P = ((double) rand() / (RAND_MAX)); //choose migration directions
    if ((P < 1.0/6.0) && (agent_row > 0)) {//Position 1
        proPosition[0] = proPosition[0] - 1;}
    else if ((P >= 1.0/6.0) && (P <1.0/3.0) && (agent_row > 0) && (agent_col < colN-1)) {//Position 2
        proPosition[0] = proPosition[0] - 1;
        proPosition[1] = proPosition[1] + 1;}
    else if ((P >= 1.0/3.0) && (P < 1.0/2.0) && (agent_col < colN-2)) {//Position 3
        proPosition[1] = proPosition[1] + 1;}
    else if ((P >= 1.0/2.0) && (P < 2.0/3.0) && (agent_row < rowN-1) && (agent_col < colN-1)) {//Position 4
        proPosition[0] = proPosition[0] + 1;
        proPosition[1] = proPosition[1] + 1;}
    else if ((P >= 2.0/3.0) && (P < 5.0/6.0) && (agent_row < rowN-1)) {//Position 5
        proPosition[0] = proPosition[0] + 1;}
    else if ((P >= 5.0/6.0) && (agent_col > 0)) {//Position 6
        proPosition[1] = proPosition[1] - 1;}
}

int CalTotal (const int rowN, const int colN) {
    int Ntotal;
    if (rowN % 2 == 0) {
        Ntotal = rowN * colN - rowN/2;
    } else {
```

```
        Ntotal = rowN * colN - (rowN+1)/2;
    }
    return Ntotal;
}

void importdata (const int N, double * dataPp) {
        ifstream ifs ("Pp.csv");
        char dummy;
        for (int i = 0; i < N; ++i){
                ifs >> dataPp[i];
                if (i < (N - 1))
            ifs >> dummy;
        }
}

int main(int argc, char **argv) {
    srand(time(NULL)); //random seeds
    random_device rd;
    mt19937 generator(rd());
    //set clock
    clock_t TIME;
    TIME = clock();

        //Initialisation
    int simuNum, sampleNum, t, T, tau, agent_row, agent_col, migPosition
        [2], proPosition[2];
    double Pm, Pp, P, avePp;
        simuNum = 1; //simulation index
    sampleNum = 2; //total simulations
    tau = 1; //time step: 1 ~ 1/12h
    t = 0; //current time
    T = 864; //end time
    Pm = 0.35;
    const int d = 24; //spacing: 1 ~ 24mum
    enum {rowN = 68, colN = 80}; //1400mum by 1900 mum
    enum {sCol1 = 29, sCol2 = 51}; //scratch width
    const int Ntotal = CalTotal(rowN,colN);
    const int Nstart = Ntotal * 0.3; //seeding condition
    cout << "Ntotal = " << Ntotal << endl;
    cout << "Nseed = " << Nstart << endl;
    static vector<vector<int> > domain(rowN, std::vector<int>(colN)); //
        simulation domain
```

```
    static vector<vector<double> > domain_Pp(rowN, std::vector<double>(
        colN)); //Pp

    ofstream file1;
    ofstream file2;
    ofstream file3;
    ofstream file4;
    ofstream file5;
    file1.open("domain0.csv");
    file2.open("domain24.csv");
    file3.open("domain48.csv");
        file4.open("domain72.csv");
        file5.open("domain96.csv");
    //Import data
    int size_data = 12790529; //size of import data
    double * dataPp;
    dataPp = new double [size_data];
    importdata(size_data, dataPp);

    //Seeding
    for (int i = Nstart; i > 0; --i) {
        agent_row = rand() % rowN;
        agent_col = rand() % colN;
        if (((rowN-agent_row) % 2 != 0) && (agent_col == colN-1)) {
            i = i + 1;
        } else if (domain[agent_row][agent_col] == 0) {
                int randPp = rand() % size_data;
            domain[agent_row][agent_col] = 1;
            domain_Pp[agent_row][agent_col] = dataPp[randPp];
        } else {
            i = i + 1;
        }
    }
    delete [] dataPp;

    //Scratch
    for (int i=0; i<rowN; ++i) {
        if ((rowN-agent_row) % 2 == 0) {
            for (int j=sCol1; j<=sCol2; ++j) {
                domain[i][j] = 0;
                domain_Pp[i][j] = 0.0;
            }
```

```
        } else {
            for (int j=sCol1; j<=sCol2-1; ++j) {
                domain[i][j] = 0;
                domain_Pp[i][j] = 0.0;
            }
        }
    }

    //Count initial cells
    int agent_N = 0;
    avePp = 0.0;
    for (int i=0; i<rowN; ++i) {
        for (int j=0; j<colN; ++j) {
            if (domain[i][j] == 1) {
                agent_N = agent_N + 1;
                avePp = avePp + domain_Pp[i][j];
            }
        }
    }
    avePp = avePp / (double)agent_N;

    //Write to files
    for (int i=0; i<rowN; ++i) {
        for (int j=0; j<colN; ++j) {
            file1 << domain[i][j];
            //file2 << domain_Pp[i][j];
            if (j < colN-1) {
                file1 << ", ";
                //file2 << ", ";
            } else {
                file1 << "\n";
                //file2 << "\n";
            }
        }
    }

    t = t + tau;
    int timestop = T / t;

    while (simuNum <= sampleNum) {
        cout << "SimuNum " << simuNum << ", ";
```

```
        cout << "N(0) = " << agent_N << ", Average Pp = " << avePp << endl;
        for (int tindex=0; tindex<timestop; ++tindex) { //check time
                int N = agent_N;
                //Migration
                for (int i=0; i<N; ++i) {
                P = ((double) rand() / (RAND_MAX)); //generate a random number
                if (P <= Pm) { //perform migration
                    //select a random site
                    int ifGhost = 0;
                    agent_row = rand() % rowN;
                    agent_col = rand() % colN;
                    while ((domain[agent_row][agent_col] != 1) || (ifGhost == 1)) { //select a random site, stop until finding an agent
                        agent_row = rand() % rowN;
                        agent_col = rand() % colN;
                    }
                    //test ghost node
                    if (((rowN-agent_row) % 2 != 0) && (agent_col == colN-1)) {
                        ifGhost = 1;
                    }
                    if (ifGhost == 0) {
                        if ((rowN-agent_row) % 2 == 0) {
                            migration1(agent_row, agent_col, rowN, colN, migPosition);
                            if (domain[migPosition[0]][migPosition[1]] == 0) {//if the new site is not taken
                                domain[agent_row][agent_col] = 0; //delete the previous site
                                domain[migPosition[0]][migPosition[1]] = 1; //move to the new site
                                domain_Pp[migPosition[0]][migPosition[1]] = domain_Pp[agent_row][agent_col]; //move the corresponding Pp
                                domain_Pp[agent_row][agent_col] = 0.0; //delete the previous Pp
                            }
                        } else {
```

```
                        migration2(agent_row, agent_col, rowN, colN, migPosition);
                        if (domain[migPosition[0]][migPosition[1]] == 0) {//if the new site is not taken
                            domain[agent_row][agent_col] = 0; //delete the previous site
                            domain[migPosition[0]][migPosition[1]] = 1; //move to the new site
                            domain_Pp[migPosition[0]][migPosition[1]] = domain_Pp[agent_row][agent_col]; //move the corresponding Pp
                            domain_Pp[agent_row][agent_col] = 0.0; //delete the previous Pp
                        }
                    }
                } else {
                    i = i - 1;
                }
            }
        }
            //Proliferation
            for (int i=0; i<N; ++i) {
            //select a random site
            int ifGhost = 0;
            agent_row = rand() % rowN;
            agent_col = rand() % colN;
            while ((domain[agent_row][agent_col] != 1) || (ifGhost == 1)) { //select a random site, stop until finding an agent
                agent_row = rand() % rowN;
                agent_col = rand() % colN;
            }
            //test ghost node
            if (((rowN-agent_row) % 2 != 0) && (agent_col == colN-1)) {
                ifGhost = 1;
            }
            if (ifGhost == 0) {
                Pp = domain_Pp[agent_row][agent_col];
                P = ((double) rand() / (RAND_MAX)); //generate a random number
                if (P <= Pp) {
```

```
                        if ((rowN-agent_row) % 2 == 0) {
                            proliferation1(agent_row, agent_col, rowN, colN, proPosition);
                            if (domain[proPosition[0]][proPosition[1]] == 0) {//if the new site is not taken
                                domain[proPosition[0]][proPosition[1]] = 1; //proliferate at the new site
                                domain_Pp[proPosition[0]][proPosition[1]] = Pp; //adopt Pp
                                agent_N = agent_N + 1; //update agent population
                            }
                        } else {
                            proliferation2(agent_row, agent_col, rowN, colN, proPosition);
                            if (domain[proPosition[0]][proPosition[1]] == 0) {//if the new site is not taken
                                domain[proPosition[0]][proPosition[1]] = 1; //proliferate at the new site
                                domain_Pp[proPosition[0]][proPosition[1]] = Pp; //adopt Pp
                                agent_N = agent_N + 1; //update agent population
                            }
                        }
                    }
                } else {
                    i = i - 1;
                }
            }
                //Write to files
            if (t == 288) { //24h
                for (int i=0; i<rowN; ++i) {
                    for (int j=0; j<colN; ++j) {
                        file2 << domain[i][j];
                        if (j < colN-1) {
                            file2 << ", ";
                        } else {
                            file2 << "\n";
                        }
                    }
                }
```

```
            }
            if (t == 576) { //48h
                for (int i=0; i<rowN; ++i) {
                    for (int j=0; j<colN; ++j) {
                        file3 << domain[i][j];
                        if (j < colN-1) {
                            file3 << ", ";
                        } else {
                            file3 << "\n";
                        }
                    }
                }
            }
            if (t == 864) { //72h
                for (int i=0; i<rowN; ++i) {
                    for (int j=0; j<colN; ++j) {
                        file4 << domain[i][j];
                        if (j < colN-1) {
                            file4 << ", ";
                        } else {
                            file4 << "\n";
                        }
                    }
                }
            }
            /*if (t == 1152) { //96h
                for (int i=0; i<rowN; ++i) {
                    for (int j=0; j<colN; ++j) {
                        file5 << domain[i][j];
                        if (j < colN-1) {
                            file5 << ", ";
                        } else {
                            file5 << "\n";
                        }
                    }
                }
            }*/
                t = t + tau; //forward time
        }

        cout << "DONE N = " << agent_N << endl;
        simuNum = simuNum + 1; //update the simulation index
```

```
        t = 0; //reset time

        if (simuNum != sampleNum+1) {
                for (int i=0; i<rowN; ++i) {
                for (int j=0; j<colN; ++j) {
                    domain[i][j] = 0;
                    domain_Pp[i][j] = 0.0;
                }
            }
            //Import data
            double * dataPp;
                dataPp = new double [size_data];
                importdata(size_data, dataPp);
            //Re-seed cells
                for (int i = Nstart; i > 0; --i) {
                        agent_row = rand() % rowN;
                        agent_col = rand() % colN;
                        if (((rowN-agent_row) % 2 != 0) && (agent_col ==
                            colN-1)) {
                        i = i + 1;
                        } else if (domain[agent_row][agent_col] == 0) {
                                int randPp = rand() % size_data;
                        domain[agent_row][agent_col] = 1;
                        domain_Pp[agent_row][agent_col] = dataPp[randPp];
                        } else {
                        i = i + 1;
                        }
                }
                delete [] dataPp;
            //Scratch
            for (int i=0; i<rowN; ++i) {
                        if ((rowN-agent_row) % 2 == 0) {
                        for (int j=sCol1; j<=sCol2; ++j) {
                                domain[i][j] = 0;
                                domain_Pp[i][j] = 0.0;
                        }
                        } else {
                        for (int j=sCol1; j<=sCol2-1; ++j) {
                                domain[i][j] = 0;
                                domain_Pp[i][j] = 0.0;
                        }
                        }
```

```
                }
            //Count cells
            agent_N = 0;
            avePp = 0.0;
                for (int i=0; i<rowN; ++i) {
                        for (int j=0; j<colN; ++j) {
                        if (domain[i][j] == 1) {
                                agent_N = agent_N + 1;
                        avePp = avePp + domain_Pp[i][j];
                        }
                        }
                }
            avePp = avePp / (double)agent_N;
            //Write to files
                for (int i=0; i<rowN; ++i) {
                        for (int j=0; j<colN; ++j) {
                        file1 << domain[i][j];
                        if (j < colN-1) {
                                file1 << ", ";
                        } else {
                                file1 << "\n";
                        }
                        }
                }
            t = t + tau;
        }
    }

    file1.close();
    file2.close();
    file3.close();
    file4.close();
    file5.close();
    TIME = clock() - TIME;
    cout << "It takes "<< (float)TIME/CLOCKS_PER_SEC <<" second(s)."<<
        endl;
    cin.get();
    return 0;
}
```
